# Dr. Nelson C. Holt

**Published:** 1918-09

**Authors:** 


### OBITUARY

Readers are requested to report promptly the death of all physicians in Western New York, or former residents of this region, or graduates of any medical school in Western New York, and to notify the families of the deceased of our desire to publish adequate Obituary notices.

**Dr. Nelson C. Holt** died in Webster July 8, age 65. He graduated from the Bellevue Hospital Medical College in 1896. He was formerly financial secretary of the Rockefeller Institute for Medical Research.

